# Study protocol: Comparison of different risk prediction modelling approaches for COVID-19 related death using the OpenSAFELY platform

**DOI:** 10.12688/wellcomeopenres.16353.2

**Published:** 2024-12-16

**Authors:** The OpenSAFELY Collaborative, Elizabeth J. Williamson, John Tazare, Krishnan Bhaskaran, Alex J. Walker, Helen I McDonald, Laurie A. Tomlinson, Sebastian Bacon, Chris Bates, Helen J. Curtis, Harriet Forbes, Caroline Minassian, Caroline E. Morton, Emily Nightingale, Amir Mehrkar, Dave Evans, Brian D Nicholson, David Leon, Peter Inglesby, Brian MacKenna, Jonathan Cockburn, Nicholas G. Davies, Will J. Hulme, Jessica Morley, Ian J. Douglas, Christopher T. Rentsch, Rohini Mathur, Angel Wong, Anna Schultze, Richard Croker, John Parry, Frank Hester, Sam Harper, Rafael Perera, Richard Grieve, David Harrison, Ewout Steyerberg, Rosalind M. Eggo, Karla Diaz-Ordaz, Ruth Keogh, Stephen J.W. Evans, Liam Smeeth, Ben Goldacre

**Affiliations:** 1 London School of Hygiene & Tropical Medicine, Keppel Street, London, WC1E 7HT, UK; 2 The DataLab, Nuffield Department of Primary Care Health Sciences, University of Oxford, Oxford, OX26GG, UK; 3 NIHR Health Protection Research Unit (HPRU) in Immunisation, London, UK; 4 TPP, TPP House, 129 Low Lane, Horsforth, Leeds, LS18 5PX, UK; 5 Nuffield Department of Primary Care Health Sciences, University of Oxford, Oxford, UK; 6 ICNARC, 24 High Holborn, Holborn, London, WC1V 6AZ, UK; 7 Leiden University Medical Center and Erasmus MC, Leiden, The Netherlands

**Keywords:** COVID-19, risk prediction, mortality, infectious disease, statistical methodology

## Abstract

On March 11th 2020, the World Health Organization characterised COVID-19 as a pandemic. Responses to containing the spread of the virus have relied heavily on policies involving restricting contact between people. Evolving policies regarding shielding and individual choices about restricting social contact will rely heavily on perceived risk of poor outcomes from COVID-19. In order to make informed decisions, both individual and collective, good predictive models are required.

For outcomes related to an infectious disease, the performance of any risk prediction model will depend heavily on the underlying prevalence of infection in the population of interest. Incorporating measures of how this changes over time may result in important improvements in prediction model performance.

This protocol reports details of a planned study to explore the extent to which incorporating time-varying measures of infection burden over time improves the quality of risk prediction models for COVID-19 death in a large population of adult patients in England. To achieve this aim, we will compare the performance of different modelling approaches to risk prediction, including static cohort approaches typically used in chronic disease settings and landmarking approaches incorporating time-varying measures of infection prevalence and policy change, using COVID-19 related deaths data linked to longitudinal primary care electronic health records data within the OpenSAFELY secure analytics platform.

## Background and aims

On March 11th 2020, the World Health Organization characterised COVID-19 as a pandemic after 118,000 cases and 4,291 deaths were reported in 114 countries ^1^. As of 2 June, towards the end of the time period considered in this study, cases were over 6 million globally, with more than 300,000 deaths attributed to the virus ^2^. In the UK, confirmed cases had reached 279,856 with 39,728 deaths ^3^.

A range of demographic factors and health conditions have been shown to be associated with poor outcomes from COVID-19, including COVID-19 related death. In the UK, the report released by Public Health England in June 2020 identified age as the strongest disparity in COVID-19 death, additionally noting disparities between males and females, and higher risks among black and minority ethnic (BME) groups ^4^. Various pre-existing conditions correlate with increased risk of poor outcomes including diabetes, respiratory disease and cancer ^5^.

On the 16 ^th^ March 2020, the UK government released advice recommending clinically vulnerable individuals to follow strict measures for reducing their social contact (social distancing). This group was broadly based on the flu at-risk group. Subsequently, on the 22 ^nd^ March 2020, a smaller ‘extremely vulnerable’ group was identified and recommended to shield.

Evolving policies regarding shielding and individual choices about restricting social contact will rely heavily on perceived risk of poor outcomes from COVID-19. In order to make informed decisions, both individual and collective, good predictive models are required. 

Clinical prediction models are widely used in many fields of medicine, including chronic disease, such as cardiovascular mortality risk, and outcomes following surgery. These models assume that the underlying context in which risk is being predicted remains relatively stable. Performance of prediction models typically deteriorates over time due to “calibration drift” ^6^. To avoid this deterioration risk models can be up-dated ^7^, either periodically or in a continuous manner (‘dynamic modelling’). Reasons for calibration drift include changes in the underlying population, improvements in general healthcare and setting-specific changes. In cases where the outcome, as here, is an infectious disease, there is an additional important component of change over time: the performance of any risk prediction model will depend heavily on the underlying burden of infection in the population of interest. While this may not affect discrimination – the ability to distinguish between cases and non-cases – it is likely to result in poor calibration, i.e. poor agreement between predicted risks and observed outcomes. One solution to this problem would be the explicit incorporation of time-varying measures of infection prevalence into the risk prediction model, which could be achieved via landmarking models ^8^.

The aim of this study is to explore the extent to which incorporating time-varying measures of infection burden and big policy changes over time improves the quality of risk prediction models for COVID-19 death in a large population of adult patients in England. The population is the general community, rather than infected people, thus the risk being predicted combines the risk of infection and the risk of dying once infected.

This aim is subtly different to that of updating models over time. In model updating, the coefficient values in the model are updated to reflect the more recent context. In our approach, (some of) the coefficients are time-varying functions, thus while their values will change over time, the parameter values of those time-varying functions will not.”

To achieve our study aim, we will compare the performance of different modelling approaches to risk prediction, including static cohort approaches typically used in chronic disease settings and landmarking approaches incorporating time-varying measures of infection prevalence and policy change, using COVID-19 deaths data linked to longitudinal primary care electronic health records data within the OpenSAFELY secure analytics platform.

### Objectives

To achieve the overarching aim of exploring the extent to which adding time-varying measures of infection burden improves the quality of risk prediction models for COVID-19 death, the following specific objectives will be addressed:

1. To develop risk prediction models for 28-day COVID-19 death within a static case-cohort design, using the following statistical approaches:
  a. Fine and Gray proportional subdistribution hazards model,
  b. Weibull model,
  c. Generalised gamma model,
  d. Royston-Parmar model.

2. To develop risk prediction models for 28-day COVID-19 death within a landmarking framework:
  a. Incorporating objective proxies of infection prevalence,
  b. Incorporating outputs from dynamic mathematical models of COVID-19 to estimate infection prevalence.

3. To evaluate model performance of each risk prediction model in predicting 28-day risk, including measures of discrimination and calibration, using internal, internal-external validation (geographical and temporal). In a subsequent study, models will be evaluated by external validation.

### Purpose of models

The overarching aim of this study is to evaluate the utility of incorporating time-varying measures of infection burden into risk prediction models for COVID-19 related death. Other risk models for COVID-19 outcomes exist, although many early models have been found to be poorly reported and at high risk of bias and over-optimism ^9^. A risk calculator has been developed to predict mortality among patients with confirmed COVID-19 in the US ^10^. Another risk calculator attempts to identify people with a heightened risk of severe complications should they become infected ^11^. The COVID-age risk score predicts risk of COVID-19 death in the general population by combining evidence from published studies ^12^. A recent model used routinely collected primary care data in the UK to predict COVID-19 mortality ^13^. Other groups, both in the UK and elsewhere, are likely to develop additional risk scores. However, to our knowledge, none of these risk scores update the person’s risk over time in reaction to changes in the underlying burden of infection.

A risk score that takes changes in the burden of infection over time into account could have a number of potential applications. Some patient groups, such as the very elderly with comorbidities, are likely to fall into the “high risk” group no matter what the prevalence of infection is. Other groups may be at higher risk, but only fall into a “high risk” categorisation when the infection prevalence is high. A risk score that provided updated risk predictions as the underlying prevalence of infection changes could allow for tailoring of GP advice, personal decisions regarding anti-COVID-19 measures and policy making. Some potential user stories are as follows:

- I am a GP and I want to know which patients are at high risk of dying from COVID-19 and when so I can advise them to reduce social contact or shield, as appropriate, during periods of particularly high risk.
- I am a patient in England and I want to know my risk of dying from COVID-19 in the near future so I can make informed decisions about whether to go in to work and whether to reduce social contact over the new few weeks.
- I am a policymaker and I want to know how risk varies so I can think about informed and transparent mechanisms for updating advice to the general public regarding social distancing, shielding and returning to work.

### Patient involvement

An important aspect of this work is to explore how patients understand the information resulting from a time-updated risk prediction and whether they find it helpful or not. In order to explore how useful and understandable patients find time-updated risk predictions we will engage with patient groups across the “risk spectrum”, recruited from existing patient participation groups from a number of different studies. We will use one-to-one interviews lasting approximately 30 minutes to discuss patients’ understanding of the risk prediction, particularly in regard to the time-updating aspect. We will develop user stories based on these interviews. We will ascertain how well patients understand the risk calculation, how accurate the prediction is and how it should be interpreted, how their prediction might change over time, and what action they should take in response to the prediction. We will explore a number of ways of presenting the information from the risk prediction and how each presentation affects the answers to the previous questions.

## Methods

### Study population, outcome and timeframe

***Population*.** The target population of interest is adults (male and female) in England between 18 and 105 years of age.

***Outcome*.** The outcome for this study is 28-day COVID-19 related death. Data are drawn from the 100-day period beginning 1 ^st^ March 2020 and ending 8 ^th^ June 2020 (inclusive).

### Model development: Data

***Database*.** Primary care records retrieved from the TPP SystmOne electronic health record system. These data include diagnoses (Read 3 CTV3), prescriptions (dm+d), basic sociodemographics and vital signs for 22 million individuals – approximately 40% of the English population.

Primary care data were linked to mortality data from the Office for National Statistics (ONS) mortality data, including COVID-19 deaths (deaths with an ICD-10 code of U071/U072 anywhere on the death certificate), and all-cause deaths (used to determine sub-study inclusion and to ascertain vital status at study entry).

The data described above will be used to develop risk prediction models and perform internal-external validation. External validation will be subsequently undertaken; details of the external data will be provided in a subsequent protocol.

***Eligibility criteria*.** The base cohort comprises adult patients (males and females, aged between 18 and 105 years) registered as of 1 ^st^ March 2020 in a general practice which employs the TPP system. Patients with missing age or a recorded age over 105 years, missing sex, or missing postcode (from which much of the household and geographic information is calculated) will be excluded. Households of greater than 10 people will be excluded, since risks experienced in institutions such as care homes are likely to be very different to those in smaller households.

### Model development: Study design

A number of different risk prediction models, predicting 28-day COVID-19 related death, will be developed. In order to do this, three different designs will be explored. (A) A case-cohort approach will be used as a computationally efficient way of exploring the traditional static cohort approach to risk prediction. (B) Landmarking will be used to explore whether additional predictive power is gained by incorporating time-varying measures of the burden of infection ^8^. In this approach, measures of infection at the beginning of a 28-day period will be used to predict 28 day risk. (C) Finally, rather than rely on information about the infection rate at the beginning of each 28-day period, the last approach fits models which update the measures of the burden of infection throughout the 28-day period, to try to better estimate the relationship between current infection prevalence and risk.

Our a priori expectations of how the three approaches will compare are as follows. The static case-cohort approaches will not incorporate any measures of the burden of infection because at the start of the cohort, infection was very low, thus including baseline measures at this point is uninformative and unlikely to increase predictive ability. We expect these static approaches to produce models which have good discrimination (can distinguish high from low risk patients well) but less good calibration (agreement between predicted and observed risks) when applied to predict absolute risk during a period of time when the prevalence of infection is very different to that used in model fitting. We expect the landmark approaches, including time-varying measures of the burden of infection, to have better calibration without losing discrimination. However, the extent of improvement in calibration will depend on how well the proxy measures of burden of infection used are able to capture the true burden of infection. The more complex landmarking approaches, in which time-varying measures of infection within the 28-day risk-prediction period are additionally accounted for, may provide better estimates of the relationship between infection prevalence and risk, but may be unstable under future predictions, due to the requirement for forecasts of infection prevalence during the period over which risks are desired for.

***Model development design A: Case-cohort study*.** The first study design will be a static case-cohort study. Follow-up will begin 1 ^st^ March 2020 and end at the first of: COVID-19 death or study end date, 8 ^th^ June 2020 (including 1 ^st^ March 2020 and 8th June in the at-risk period). The outcome is COVID-19 related death. The sub-distribution hazard for COVID-19 death is estimated by not censoring participants if they experience the only competing event (death due to non-COVID causes) ^14^.

Due to large numbers of eligible patients (~17 million), a sampling approach will be adopted, using weights to account for the sampling in analysis as described in the analysis section. The analysis sample will include all cases of COVID-19-related death and a random sample of the eligible patient population (the ‘subcohort’, largely comprising non-cases but likely to contain some cases by chance) ^15, 16^. The strongest predictor of COVID-19 related death is age ^5^, so sampling will be stratified by age-group. Sampling fractions will be: 0.01 in the age-group 18-<40, 0.02 in 40-<50, 0.02 in 50-<60, 0.025 in 60-<70, 0.05 in 70-<80, 0.13 in 80+ years. The sampling fractions have been chosen to result in a subcohort of at least 40:1 of non-cases to cases overall. This sampling strategy was selected because information relating to risk tends to lie largely in participants who experience the outcome, which is a very small fraction of the overall population here. Random sampling would greatly reduce the information available with which to predict risk. Case-cohort sampling, with the weights we propose to use (Barlow), has been shown to lead to very good agreement of absolute risks and measures of calibration and discrimination in comparison with use of the full underlying cohort ^17^.

**Figure 1.**
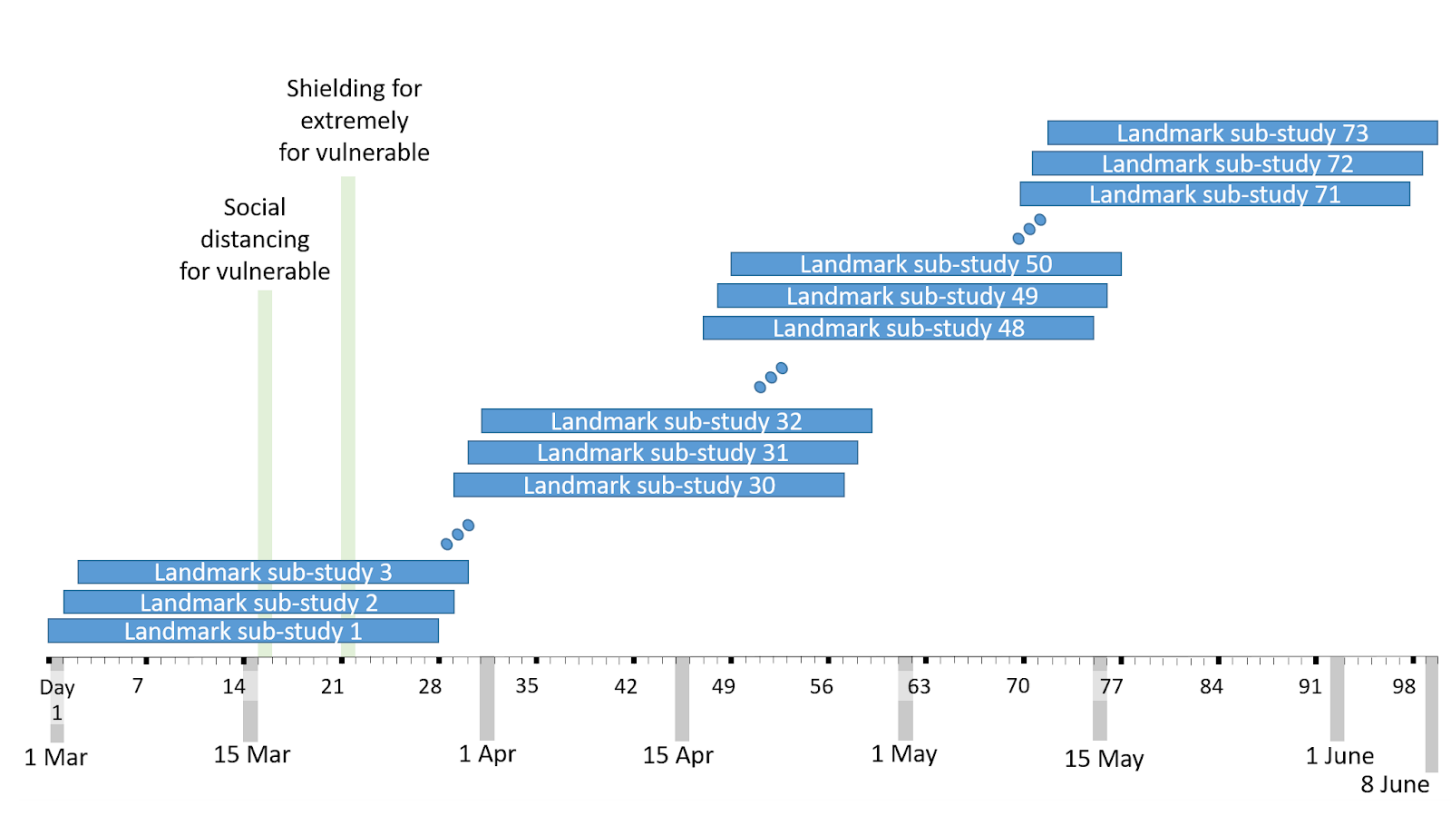
Schematic of landmarking sub-studies.

***Model development design B: Landmark case-cohort sub-studies*.** This approach comprises a series of 73 overlapping sequential sub-studies. From 1 ^st^ March 2020, 73 sub-studies will be defined, starting on 0, 1, 2, 3, 4…, 72 days after March 1 ^st^. Each sub-study will have a duration of exactly 28 days. The last sub-study begins 12 ^th^ May and ends 8 ^th^ June 2020. Figure 1 shows a schematic of the sub-studies.

All patients from the base cohort who remain alive at the end of the day prior to the sub-study entry date will be eligible to participate in that sub-study. Follow-up will start at sub-study entry date and end at the first of COVID-19 related death or sub-study end date (28 days after sub-study entry date). As for design A, participants will not be censored at deaths due to other causes.

Each sub-study will have a case-cohort design. All eligible patients who experience a COVID-19 related death during the 28-day sub-study period will be included as cases. An age-stratified random sample of sub-study eligible patients (the sub-study sub-cohort) will additionally be selected, with age-group specific sampling fractions equal to 1/70 of the sampling fractions for approach A (e.g. 0.01/70 = 0.00014 in the age-group 18-<40) to give approximately the same number of cases and non-cases overall in the model development samples for approach A and B. Each resulting sub-study sub-cohort will largely comprise non-cases but will include some cases by chance. The 73 sub-studies will be combined for analysis, as described below.

***Model development design C: Daily landmark case-cohort sub-studies*.** The third approach differs from the second by incorporating information about changes in the infection prevalence during the 28-day sub-study duration. Two slightly different designs will be used within approach C.

The first (approach Ci) will use the landmark case-cohort sub-studies described for design B, but analysed in a way that additionally accounts for changes in infection prevalence during the 28-day sub-study period.

The second (approach Cii) will also be a series of stacked sub-studies. In this case, 100 sub-studies will be formed, the first starting on 1 ^st^ March 2020 and the last starting on 8 ^th^ June 2020. In contrast to approaches B and Ci, the duration of these sub-studies will be a single day. Each sub-study will include all cases (COVID-19 related deaths) that occur on that day and a random sample of non-cases who remained alive by the previous day. As above, sampling will be stratified by age-group, with sampling fractions equal to 1/1000 of the sampling fractions for approach A (e.g. 0.01/100 = 0.0001 in the age-group 18-<40). The outcome will be the binary outcome of whether or not the sub-study participant experienced a COVID-19 related death on that day.

Approach Cii also requires information about the daily rate of death due to other causes. This will be estimated in a second case-cohort sample, comprising a sampling fraction of 0.3 of all non-COVID-19-related deaths on each day, to provide approximately 8,000 deaths due to other causes. Age-stratified sampling will be used to sample patients who do not die of non-COVID-19-related causes on that day, with sampling fractions equal to 1/100 of the sampling fractions for approach A (e.g. 0.01/100 = 0.0001 in the age-group 18-<40). For both COVID-19 related death and other death, the sampling fractions have been chosen to give a ratio of approximately 40 non-cases to cases.

### Model development: Candidate predictors

***Individual characteristics*.** The outcome, COVID-19 related mortality, is the result of a number of processes: exposure, infection and then death following infection. Because of this, there are a range of mechanisms driving associations between patient characteristics and the outcome. We selected candidate predictors based on known or plausible associations with exposure to COVID-19 infection, risk of respiratory tract infection or severity of illness, and factors associated with healthcare access or level of care, as shown in Table 1.

**Table 1.** Rationale for selection of candidate predictors.

Category	Example predictors
Factors associated with exposure to infection	Age, sex, ethnicity, household size, deprivation, region
Risk factors for infection (given exposure) or severity of COVID-19 infection	Smoking, obesity, underlying health conditions known to be risk factors for severe respiratory tract infection, risk factors for thrombosis
Barriers to healthcare access or level of care	Markers of frailty, terminal illness, ethnicity, age, mental health status

Candidate predictor variables are described below. The codelists used to define these variables are listed in the *Extended data* ^18^.

Demographic measures included as candidate predictors are: age (continuous); sex (male or female); ethnicity (eight categories: White, Indian, Pakistani, Bangladeshi/Other Asian, African/Other black, Carribean, Chinese, Mixed/Other); deprivation (quintile of the index of multiple deprivation (IMD) derived from the patient’s postcode at lower super output area level). Two household measures will be included: the number of adults living in the household (continuous) and whether or not children aged up to 12 years are living in the household (yes/no). The region (seven regions of England: South West; South East; London; East; Midlands; North West; North East, Yorkshire and the Humber) and whether the individual lives in a rural or urban area will be included.

Lifestyle characteristics to be included as candidate predictors are: obesity category and smoking status. Obesity was grouped using categories derived from the World Health Organisation classification of Body Mass Index (BMI; kg/m ^2^): underweight <18.5 kg/m ^2^; obese I 30-34.9; obese II 35-39.9; obese III 40+; or no evidence of obesity or being underweight, with BMI ascertained from weight measurements within the last 10 years, restricted to those taken when the patient was over 16 years old. Smoking status was grouped into evidence of current smoking in the last 18 months, former and never smokers.

A number of comorbidities will also be included as candidate predictors. These are defined through combinations of clinical measurements, prescriptions, and recorded diagnoses. Blood pressure (in a measurement taken in the last 18 months) was grouped into: high II (systolic blood pressure (SBP) >= 140 mmHg or diastolic blood pressure (DBP) >= 90 mmHg), high I (SBP 130-<140 or DBP 80-<90), elevated (SPB 120-<130 and DBP <80) or normal (SBP<120 and DBP < 80). Where the two measures (SBP and DBP) differed, the higher (worse) class was taken. Other comorbidities included: diagnosed hypertension; chronic cardiac disease including chronic heart failure, ischaemic heart disease, and severe valve or congenital heart disease likely to require lifelong follow up; atrial fibrillation; surgery for peripheral arterial disease or lower limb amputation; prior deep vein thrombosis or pulmonary embolism; diabetes (additionally using HbA1c within last 15 months to determine level of HbA1c control, grouped into <58 mmol/mol (good control), >=58 mmol/mol (poor control) and no recent measure); stroke; dementia; and other neurological conditions (motor neurone disease, myasthenia gravis, multiple sclerosis, Parkinson's disease, cerebral palsy, quadriplegia or hemiplegia, malignant primary brain tumour, and progressive cerebellar disease).

Candidate predictors related to respiratory disease are: asthma (grouped by use of oral corticosteroids as an indication of severity, with two or more prescriptions in the last year taken to indicate severe asthma); cystic fibrosis and associated diseases such as primary ciliary dyskinesia; and other respiratory disease. Candidate predictors related to malignancy are: haematological malignancies (considered separately from other cancers to reflect the immunosuppression associated with haematological malignancies and their treatment) and non-haematological malignancy, each grouped according to time since diagnosis (<1 year, 2-<5 years, 5+years). Candidate predictors related to liver and kidney function are: liver disease; solid organ transplant (any); dialysis, for patients who have not since had a kidney transplant; and kidney function (ascertained from the most recent serum creatinine measurement taken in the last 5 years excluding the most recent fortnight, where available, converted into estimated glomerular filtration rate (eGFR) using the Chronic Kidney Disease Epidemiology Collaboration (CKD-EPI) equation), with reduced kidney function grouped into no evidence of kidney impairment (no creatinine measurement or eGFR>=60 mL/min/1.73m ^2^), stage 3 (eGFR in range 30-<60 mL/min/1.73m ^2^) and stage 4–5 (<30 mL/min/1.73m ^2^). Patients with a history of kidney dialysis or kidney transplant will be included in the category representing stage 4–5.

Other candidate predictors include: common autoimmune diseases including rheumatoid arthritis (RA), systemic lupus erythematosus (SLE) or psoriasis; asplenia (splenectomy or a spleen dysfunction, including sickle cell disease); other immunosuppressive conditions including a condition inducing permanent immunodeficiency ever diagnosed, or aplastic anaemia or temporary immunodeficiency recorded within the last year; inflammatory bowel disease; and HIV. The final candidate predictors are: learning disability, including Down’s syndrome; serious mental illness; and fragility fracture in the last two years for patients aged 65 or above.

***Time-varying measures of disease burden*.** Three different sources of measures of burden of disease will be considered. In each case, measures will be obtained daily.

- Estimates of the force of infection from dynamic disease modelling. These will be obtained by region and by 5-year age-group. These estimates take into account the infection prevalence, the way in which different age-groups interact with each other and the proportion of the population who are susceptible.
- COVID-19 related A&E attendances among our database within each Sustainability and Transformation Partnership (STP, used as a measure of local geographic area). STPs in which insufficient data is available within our database to obtain reliable rates will be combined with a geographically adjacent STP. We will smooth this measure by taking the mean daily rate over the last 7 days, on each day for which a measure is required. A&E attendances is likely to be an imperfect proxy for infection prevalence since it is likely to lag behind true prevalence of infection.
- Suspected COVID-19 cases as recorded in GP records by STP, with smaller STPs combined with a geographically adjacent STP. CTV3 Codes XaaNq, Y20cf, Y211b, Y22b7 and Y22b8 will be taken to indicate a suspected case (see *Extended data*) ^18^. We will take the mean rate over the last 7 days. Suspected cases may also lag behind the true infection prevalence. Moreover, there may be differences over time in how and when people visit their GP, so changes over time may be less likely to reflect true changes in infection prevalence than measures of more severe COVID-19, such as A&E attendances.

***Omitted predictors*.** We have chosen not to include a measure of geographical region in our models, despite clear differences in COVID-19 outcomes by region. The rationale for this is that regional differences in COVID-19 related death are expected to be explained largely by differences in the burden of infection and geographical differences in comorbidities. By explicitly including these factors, remaining differences in region should be minimised. The omission of region would also facilitate the application of resulting models to regions other than those used in the model development process, although validation in those regions would be required to ascertain the performance of the models.

We considered attempting to tease out the effect of key government policies, such as the shielding policy, on COVID-19 related death through their effects on the burden of infection. However, the timing of such policies is inextricably linked to periods in which infection rates are highest, thus statistical models such as those described in this document are not able to separate reductions due to shielding from the overall higher level of infection present when shielding was imposed.

### Model development: Statistical analysis

While the focus of this study is on the impact of explicitly modelling the changing burden of infection over time, some of this impact may occur through changes in the variables that are selected to be in the model. Variables that strongly predict risk in models that do not account for the changing burden of infection over time may have a weaker, or non-existent, relationship with risk once this aspect is explicitly modelled. Therefore, variable selection is intrinsically bound up in our comparison of interest. Below, we describe our variable selection strategy.

***Variable selection A: Case-cohort study*.** Variable selection will be performed, for approach A, within a 4% random sample of the whole eligible cohort. The sampling fraction was chosen as the largest fraction possible while keeping computational resources within the limits that will be available for this project. We anticipate this resulting in a variable selection sample of around 500,000 individuals, with at least 250 COVID-19 related deaths.

A Poisson model will be used for variable selection, using the whole 100-day period, with follow-up time accounted for via an offset term. Predictor variables will be selected from the pool of candidates, as listed above, by a lasso, with a penalty parameter chosen by 3-fold cross-validation. Continuous variables (age and number of people in the household) will be standardised. Restricted cubic splines of the standardised variables will be included. Standardised age and sex will be forced into all models. The following variables will be considered (by the lasso) for inclusion: rural/urban classification; deprivation; ethnicity; obesity; smoking status; blood pressure category; diagnosed hypertension; diabetes; chronic cardiac disease; atrial fibrillation; surgery for peripheral arterial disease or lower limb amputation; deep vein thrombosis or pulmonary embolism; stroke, dementia, other neurological conditions; asthma; cystic fibrosis; other respiratory disease; haematological malignancies; non-haematological malignancies; liver disease; dialysis; solid organ transplant; kidney function; common autoimmune diseases; asplenia; other immunosuppressive conditions; inflammatory bowel disease; HIV; learning disability; serious mental illness; fragility fracture; the presence of children in the household; household size (spline terms); and age (spline terms). Interactions between age (linear effect) and sex and each other candidate variable will also be included for consideration. Variables with non-zero coefficients will be included in the subsequent models.

***Variable selection B and C: Landmarking study*.** Variable selection will be undertaken separately for each measure of burden of infection. For each measure, we will first select the functional form of the time-varying measure of the burden of infection. This will be done in a logistic model within the stacked case-cohort sub-studies, unadjusted for any other variables comparing different forms of the time-varying measures using the Akaike information criterion (AIC). For each time-varying measure of the burden of infection (estimated force of infection, A&E attendance rate or suspected case rate), models considered will consider for inclusion: the current measure (evaluated as of day 0 of the relevant sub-study); the log of the current measure; the coefficients from a quadratic model of the measure fitted to the previous three weeks of data, expressed in relation to the current measure; and polynomial terms of the current measure. Considering these non-linear and quadratic coefficients for inclusion attempts to capture the direction of change in the burden of infection.

Once the functional form of the time-varying measure of the burden of infection has been selected, variable selection will be performed using a random sample from the whole cohort. Three non-overlapping 3% random samples of individuals from the whole eligible cohort will be selected. Data from the 28-day period 1st March – 28th March (inclusive) will be used for individuals in the first random sample; data from 6th April – 3rd May (inclusive) for individuals in the second; and data from 12th May – 8th June for the third. Taking 28-day blocks from three non-overlapping periods, rather than all 73 landmark sub-studies for this step minimises effects of repeatedly including the same individuals on the variable selection process. We anticipate this resulting in more than 500,000 individuals and at least 300 COVID-19 related deaths.

Variable selection will be performed within the three random samples, stacked to form one dataset, using a logistic regression lasso. The candidate pool will be as above, with continuous variables and interactions treated as above. The chosen functional form of the relevant time-varying measure of the burden of infection will be forced into the models. Approach B will use the resulting selected set of variables. Approach C will use the same covariates as for approach B but the functional form of the time-varying measure of the burden of infection will be investigated separately within the one-day stacked sub-studies.

***Sample size considerations*.** To explore how many parameters can reasonably be included in the prediction models, we undertook a range of sample size calculations following Riley *et al.* ^19^ The sample size calculations are undertaken for a binary outcome and assume a logistic regression model is being fitted. 

We assume that the overall prevalence of COVID-19-related death in the whole cohort is 0.000471. The maximum value the Cox-Snell R-squared value can take is bounded above by a function of the outcome prevalence, here 0.00812. We set the maximum acceptable difference in apparent and adjusted R-squared to 0.05 and the margin of error in the intercept estimation to 0.05. The number of candidate predictor parameters is set at 60 and 120. We assume the model will explain 10%, 20%, 50% and 70% of the variability in the outcome, to obtain a range of required sample sizes.

Table 2 shows the estimated sample sizes required. The anticipated sample sizes should provide sufficient power to estimate up to around 60 parameters, assuming we will be able to explain at least 10% of the variability. For 120 parameters, we would need to be able to explain 20% of the variability in order to have sufficient power with our proposed random sample.

**Table 2.** Estimated sample sizes required for models with 60 and 120 parameters.

Proportion of variability explained	Required sample size	Total events required	Events required per predictor
**To estimate 60 parameters**			
10%	664,834	313	5.21
20%	332,267	157	2.61
50%	132,727	63	1.04
70%	119,414	57	0.94
**To estimate 120 parameters**			
10%	1,329,668	626	5.21
20%	664,534	313	2.61
50%	265,454	125	1.04
70%	238,827	113	0.94

***Missing data*.** A large number of the candidate predictors will be fully observed, in the sense that an absence of a diagnosis is taken to indicate the absence of disease, as is typically assumed in electronic health record research. While this may lead to issues with misclassification and subsequent interpretation, this does not manifest itself in a missing data problem. Missing data will arise in some demographic variables and clinical measurements, with the later predominantly used to determine severity of certain conditions. The predictors that are expected to have missing data (with anticipated missing rates in brackets) are: ethnicity (~25%), BMI (~20%), Smoking (~5%), hba1c (~20% of patients with diabetes), and kidney function (missingness likely in serum creatinine measurement). Our previous analyses in these data suggested that the missingness mechanism for ethnicity may be somewhat missing not at random in one region, but little evidence against missing at random in the other regions.

For the main comparisons between risk prediction modelling strategies, a complete case approach will be used for ethnicity, restricting the analysis and validation steps to the sub-population in which ethnicity is measured. Patients with missing BMI will be assumed non-obese and patients with no smoking information will be assumed non-smokers, on the assumption that smoking and obesity, if present, are likely to be recorded. Patients with no serum creatinine measurement will similarly be included in the “no evidence of poor kidney function”. Patients with diabetes but no Hba1c measurement will be included in a separate “diabetes, no Hba1c” category. Pre-specified exploratory analyses (described below) will explore the application of multiple imputation for missing data.

***Model estimation A: Case-cohort study*.** The following statistical models will be fitted, with time since 1 ^st^ March 2020 as the timescale: Cox Fine and Gray proportional subdistribution hazards model, Weibull, Royston-Parmar, Generalised gamma. The Royston-Parmar model is a survival model that flexibly models the baseline log cumulative hazard function using restricted cubic splines. We will fit this model with 5 degrees of freedom, resulting in 4 knots spread evenly across the quintiles of the uncensored log survival times (at 20, 40, 60 and 80). If the Royston-Parmar and Generalised gamma models fail to converge, we will omit these models.

Barlow weights will be used to account for the case-cohort design ^15, 16^. Subcohort participants will be weighted by the inverse of the sampling fraction. Cases (COVID-19-related deaths) enter the risk set on the day of death with a weight of 1. Prior to that: cases not in the subcohort receive a weight of zero; cases in the subcohort receive a weight of the inverse of the sampling fraction. Robust standard errors will be used.

The variables selected via the lasso procedure above will be included in the models. Note that these models do not include any time-varying covariates or time-varying measures of burden of infection.

***Model estimation B: Landmarking sub-studies*.** Data from all 73 sub-studies will be stacked to form one analysis dataset, with a variable indicating the sub-study (k=1,2,…,73). Barlow weights with robust standard errors will be used. A Poisson, Weibull, and logistic model will be fitted.

The variables selected via the lasso procedure above will be included in the models. Covariates (e.g. patient comorbidities) and time-varying measures of the burden of infection will be evaluated at day 0 of the relevant landmarking sub-study (the day before follow-up begins). 

***Model estimation C: Daily landmarking sub-studies*.** Two approaches will be used. In the first (approach Ci), landmarking will be used as above. Within each 28-day sub-study, time will be split into the four weeks. A Poisson model will be fitted, similar to those described for approach B, additionally allowing the measures of infection prevalence and the shielding indicators to change each week. Patients dying due to non-Covid-19 related causes will not be censored in this approach.

In the second approach (Cii), a series of 1-day studies will be stacked to form a single analysis dataset. A Poisson model, incorporating measures of prevalence of infection (as selected by the lasso process for approach B), will be fitted to estimate the daily rate of the outcome, COVID-19 related death. Non-cases will be weighted by the inverse of the sampling fraction and cases by 1, with robust standard errors. In a similar way, a Poisson model will be fitted to estimate the daily rate of mortality due to non-COVID-19-related causes conditional on the same set of predictor variables, but without the measures of the burden of infection, weighted according to the inverse of the sampling fractions. Risk of 28-day COVID-19 related death will be estimated using a sum of the daily estimated risk of COVID-19 death multiplied by the estimated daily probability of surviving from other causes.

### Model validation

**Figure 2.**
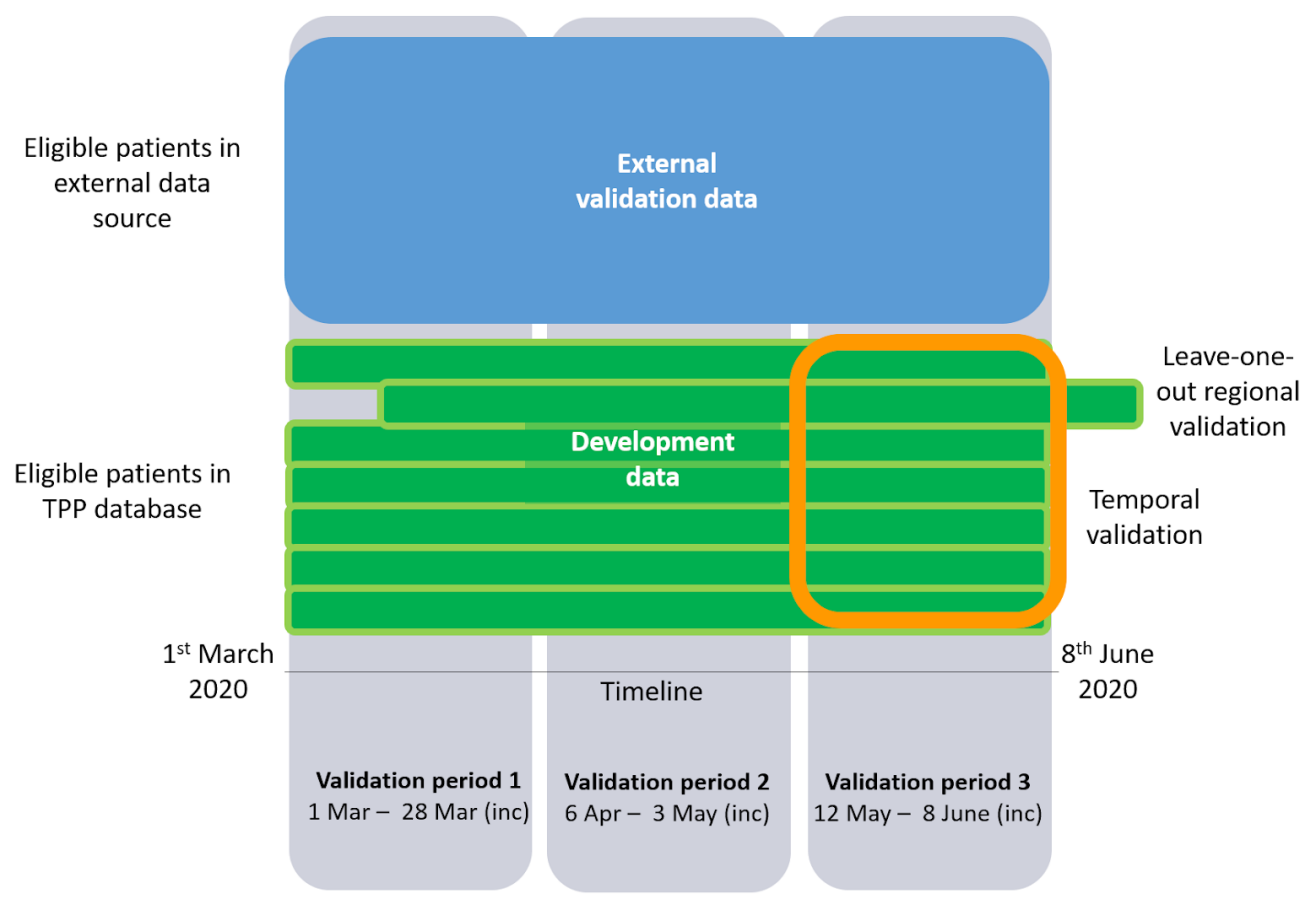
Schematic of the different datasets to be used to develop and validate the models.

The outcome being predicted is 28-day COVID-19-related mortality, so validation will be undertaken in 28-day periods. Three validation periods will be considered: validation period 1 will run from 1st March – 28th March, validation period 2 will run from 6th April – 3rd May, validation period 3 will run from 12th May – 8th June. For all date ranges, the start and end date will be included in the follow-up time to give periods of 28 full days. These validation periods have been chosen to cover periods of higher and lower infection prevalence, within the period of time used for model development. Figure 2 shows a schematic of the different data sources to be used to develop and validate the models.

For each model, measures of model performance will be obtained using:

- Internal validation (validation periods 1, 2 and 3)
- Geographical internal-external validation (validation periods 1, 2 and 3)
- Temporal internal-external validation (validation period 3)

Models to be evaluated are shown in Table 3.

**Table 3.** Models proposed for evaluation.

Model	Approach				Model for COVID-19-related death						Proxy for burden of infection			
	A	B	Ci	Cii	Logistic	Fine & Gray	Poisson	Weibull	Royston- Parmar	Generalised gamma	None	Modelling output	A&E attendances	Suspected GP cases
1	X					X					X			
2	X							X			X			
3	X								X		X			
4	X									X	X			
5		X			X							X		
6		X			X								X	
7		X			X									X
8		X					X					X		
9		X					X						X	
10		X					X							X
11		X						X				X		
12		X						X					X	
13		X						X						X
14			X				X					X		
15			X				X						X	
16			X				X							X
17				X			X					X		
18				X			X						X	
19				X			X							X

In addition, for the static models (approach A), model performance will be evaluated over the whole 100-day period used to fit the models.

For the geographical internal-external validation, a leave-one-out approach will be used, omitting all patients from one geographical region in turn, performing the model selection and fitting the model in the sub-sample excluding that region, and then using the fitted model to make predictions for the patients in the omitted region. This will be repeated for each of seven regions. Model performance measures will be averaged over the seven sub-analyses. For the temporal internal-external validation, the data will be split into two time-periods: 1 March 2020 until 11 May and 12 May until 8 June 2020.

***Risk prediction*.** When predicting 28-day risk in a particular validation period, all patients who remain alive by the end of the day prior to the start of the validation period will be included. Characteristics will be assessed on the day prior to the start of the validation period. For validation periods 2 and 3, therefore, new comorbidities not present at baseline may have occurred. 

Risks of 28-day COVID-19-related death will be predicted using each model. Model performance will be assessed by comparing observed outcomes, 28-day COVID-19-related death, to the predicted risk. Note that since there is no censoring, we observe the outcome for all patients. 

***Measures of model performance*.** Discrimination – the ability to distinguish between cases and non-cases – will be assessed by Harrell’s C-statistic. The Brier Score will be used as a measure of overall model performance ^15^. Calibration – the agreement between observed outcomes and predictions will be assessed in three ways. First, mean calibration will be assessed by comparing mean predicted risk with mean observed risk. Second, the calibration intercept and slope will be used to assess whether models over- or under-estimate risk or provide overly extreme or modest risk estimates. Third, the Hosmer-Lemeshow goodness-of-fit p-value will be calculated, comparing observed and expected numbers of events within deciles.

While case-cohort samples will be used for model fitting to reduce computational burden, measures of model performance will be calculated on the full cohort validation samples. For approach A, when evaluating model performance for the whole 100-day period, the whole cohort will be used if possible. If computational problems are encountered, the 100-day case-cohort sample will be used, with the calibration measures modified to account for the case-cohort design. In all cases, the outcome will be treated as a binary outcome for the purposes of model evaluation.

***Model comparison*.** Comparison will be made between models in terms of model performance - calibration, discrimination and overall accuracy – in the internal validation and in the geographical and temporal internal-external validations.

***External validation*.** This protocol focuses on the model development, internal and internal-external validation of the models. Subsequently, external validation will be undertaken using a UK data source external to the one used for the model development.

Covariates will be defined in an analogous way as for the TPP data, insofar as is possible, allowing for differences in coding schemes used in primary care systems across the UK. 

The same validation periods will be used, as shown in Figure 2. Models developed in the current work will be used to predict risk in the external data within each validation period. Measures of model performance, as described for the internal validation, will be obtained. 

### Pre-specified exploratory analyses

A number of pre-specified exploratory analyses will be conducted. These will be used to explore the impact of modelling decisions made about and to contextualise interpretation of the models developed above.

First, a “parsimonious” model will be proposed, informed by the selected predictor set and clinical judgement. Variables thought to act on the risk of COVID-19 related death via similar mechanisms will be grouped into coarser categories. This may involve re-introducing subcategories that were removed in the lasso above, by virtue of belonging to a coarser clinical grouping. The performance of this parsimonious model will be compared to an analogous model using the lasso-selected set of variables. Conversely, we will assess a “richer” model, which includes additional parameters to those selected by the lasso. Specifically, if one or more dummy variables in a multi-category variable is selected for inclusion, the whole variable will be included in the final model. Similarly, interactions with the whole categorical variable will be included if any dummy variable is selected within an interaction term. We will take the best performing model(s) from the main evaluation and compare model performance of the selected model with the analogous parsimonious and richer models.

Second, if our results suggest that adding the time-varying measures of infection burden do enhance predictive accuracy, we will directly assess the extent of this by comparing measures of model performance of the landmarking approach B, including the best performing time-varying measures of burden of infection, with the same approach but omitting all such time-varying measures (and re-doing variable selection without these variables).

Third, we will explore the extent to which region adds to model performance of the best fitting models in approach A and B, to explore whether adding the time-varying measures of burden of infection remove or reduce apparent regional differences.

Fourth, it is possible that covariate information is more complete or more up-to-date for patients hospitalised with COVID-19, which could bias the estimates from the time-updated models. Therefore, we will repeat one of the models without updating covariates (i.e. using covariates as defined at day 0 of the base cohort), in order to assess the impact of this decision.

Fifth, we have arbitrarily decided to use the previous three weeks of data to model patterns of change in time-varying measures of the burden of infection, summarised through a quadratic model. We will explore other modelling approaches, comparing models using the Akaike information criterion. Specifically, we will take one model from approach B and explore the effect of changing the three-week look-back period to two or four weeks. We will compare the quadratic model with other models, such as a cubic or fractional polynomial.

Finally, the best fitting model will be refitted following multiple imputation, imputing all covariates above with missing data, with the subsequent model re-validated in the same way as the previous models. The multiple imputation process will be via chained equations, including the binary outcome, the Nelson-Aalen estimate of the cumulative hazard, all predictors and interaction terms in the model ^20, 21^. 10 imputed datasets will be created within the model development dataset with models fitted in each dataset and combined using Rubin’s rules. To validate the models, multiple imputation will be separately undertaken in each validation dataset, including the outcome as above, with validation measures calculated in each imputed dataset and combined using Rubin’s rules. Five imputed datasets will be created in each validation dataset, due to computational considerations. This imputation attempts to quantify model accuracy that would be achieved when implementing in data that has no missingness.

### Ethics and information governance

NHS England is the data controller; TPP is the data processor; and the key researchers on OpenSAFELY are acting on behalf of NHS England. This implementation of OpenSAFELY is hosted within the TPP environment which is accredited to the ISO 27001 information security standard and is NHS IG Toolkit compliant; patient data has been pseudonymised for analysis and linkage using industry standard cryptographic hashing techniques; all pseudonymised datasets transmitted for linkage onto OpenSAFELY are encrypted; access to the platform is via a virtual private network (VPN) connection, restricted to a small group of researchers, their specific machine and IP address; the researchers hold contracts with NHS England and only access the platform to initiate database queries and statistical models; all database activity is logged; only aggregate statistical outputs leave the platform environment following best practice for anonymisation of results such as statistical disclosure control for low cell counts. The OpenSAFELY research platform adheres to the data protection principles of the UK Data Protection Act 2018 and the EU General Data Protection Regulation (GDPR) 2016. In March 2020, the Secretary of State for Health and Social Care used powers under the UK Health Service (Control of Patient Information) Regulations 2002 (COPI) to require organisations to process confidential patient information for the purposes of protecting public health, providing healthcare services to the public and monitoring and managing the COVID-19 outbreak and incidents of exposure. Taken together, these provide the legal bases to link patient datasets on the OpenSAFELY platform. This study was approved by the Health Research Authority (REC reference 20/LO/0651) and by the LSHTM Ethics Board (reference 21863).

### Dissemination

All data were linked, stored and analysed securely within the OpenSAFELY platform ( https://opensafely.org/). Detailed pseudonymized patient data are potentially reidentifiable and therefore not shared. We rapidly delivered the OpenSAFELY data analysis platform without prior funding to deliver timely analyses on urgent research questions in the context of the global COVID-19 health emergency: now that the platform is established we are developing a formal process for external users to request access in collaboration with NHS England. Details of this process will be published shortly on the OpenSAFELY website.

Data management was performed using Python 3.8 and SQL, with analysis carried out using Stata 16.1 and Python. All code is shared openly for review and reuse under an MIT open license. All code for data management and analysis will be archived online, once the analyses described in this document have been undertaken, at https://github.com/opensafely/risk-prediction-research. All clinical and medicines codelists are openly available for inspection and reuse at https://codelists.opensafely.org/.

### Study status

We are currently finalising the data management and wrangling for this study. We have defined the required study population and created the variables needed. Data checks are currently underway. We will begin the analysis described herein this week (12 October 2020).

## Discussion

This protocol has detailed the methods to be used for our study which aims to explore the extent to which incorporating time-varying measures of infection burden over time improves the quality of risk prediction models for COVID-19 death. The study will use COVID-19 deaths data linked to longitudinal primary care electronic health records data within the OpenSAFELY secure analytics platform. Importantly, this study will be the first to explore how to optimally incorporate time-varying measures of the burden of infection. If the incorporation of these data substantially improves the predictive ability of risk prediction models in COVID-19, this will have important implications for best practice in risk prediction in this area.

This protocol has been written in the light of relevant guidelines, including the TRIPOD reporting guidelines and the PROBAST risk of bias guidelines for prediction models ^22, 23^.

The planned study has a number of limitations. Importantly, the outcome is COVID-19-related death among the general population, thus the outcome reflects the combined processes of becoming infected and dying. It is not possible to separate the risk prediction into those two component parts. Second, the outcome relates to short-term mortality and does not account for quality of life or life years lost. Third, the use of primary care data allows a very large cohort to be used to address this question, but these data are not perfect. Some data are missing, other information is misclassified or imperfectly measured. The interpretation of variables, as measured in routinely collected primary care electronic health records, may not be the same as the answer you might get from the same patient if using a questionnaire, for example. Fourth, we are using candidate predictors only from primary care data, thus the information available on some conditions, such as cystic fibrosis or HIV control, is incomplete or not available. This may slightly dilute estimates, in comparison to those we would see if we had more detailed data on these conditions. Fifth, if we find that adding time-varying measures of burden of infection enhances predictive ability, model performance could change over time if the way our proxies for the burden of disease are measured change. For example, if we found A&E attendances for COVID-19 added to the predictive power of our modes, this may change if people started visiting A&E for much less severe cases of COVID-19.

Machine learning models are increasingly being used to derive risk predictions in heath context. Common approaches include classification trees, random forests, artificial neural networks and support vector machines. In this protocol, the contribution of machine learning techniques is restricted to variable selection procedures, via the lasso. For model estimation, we chose to use a more statistical approach, relying on maximum likelihood regression modelling. This is because, despite much enthusiasm for machine learning approaches to risk prediction, there is little evidence to suggest that they perform better than traditional statistical models in clinical contexts ^24^.

In conclusion, we have outlined a protocol to explore whether the addition of time-varying measures of the burden of infection substantially improves performance of models predicting short-term COVID-19-related death. The resulting models may allow general practitioners, policy makers and individuals to make informed decisions regarding social contact and shielding behaviours accounting for the changing nature of the epidemic.

## Data availability

### Underlying data

No underlying data are associated with this article.

### Extended data

Zenodo: Extended data: Codelist details for risk prediction protocol for COVID-19 related death

https://doi.org/10.5281/zenodo.4073340 ^18^.

This project contains the following extended data:

- Extended_data_Risk_prediction_protocol_8102020.docx (Codelist details for risk prediction protocol for COVID-19 related death)

Data are available under the terms of the Creative Commons Attribution 4.0 International license (CC-BY 4.0).

## Author information

Authors Liam Smeeth, Ben Goldacre jointly supervised this work, while Elizabeth J Williamson, John Tazare, Krishnan Bhaskara and Alex J Walker contributed equally.
