# Supplementary material for: Study protocol: Comparison of different risk prediction modelling approaches for COVID-19 related death using the OpenSAFELY platform
Source: Wellcome Open Res. 2024 Dec 16;5:243. Originally published 2020 Oct 15. [Version 2] doi: 10.12688/wellcomeopenres.16353.2 (PMC11809169; doi:10.12688/wellcomeopenres.16353.2)
Supplement: Peer review history

# Reviewer report: Reviewer response for version 2

**DOI:** 10.21956/wellcomeopenres.25956.r115875

**Published:** 2025-01-06

**Authors:** Niamh Meagher

**Affiliations:** 1 Department of Infectious Diseases, The Peter Doherty Institute for Infection and Immunity (Ringgold ID: 534133), Melbourne, Victoria, Australia

This paper details the protocol for development, validation and comparison of statistical prediction models for risk of COVID-19 mortality using a large UK health database (OpenSAFELY). The aim is to compare the performance of models that do and do not account for time-varying measures of disease prevalence. The authors hypothesis that inclusion of time-varying measures will ultimately produce better performing models that are less impacted by calibration drift.

 This protocol provides extensive methodological detail, covering all areas relevant to the conduct of the study - the source data and participant eligibility to model development, variable selection, and model validation. The authors have also provided detailed responses to the previous reviewers to address the key concerns raised at that time, and my understanding is that the findings from this work has subsequently been published. As such, I don’t have any recommendations for changes to this existing protocol and I commend the authors on a novel and interesting study.

Is the study design appropriate for the research question?

Yes

Is the rationale for, and objectives of, the study clearly described?

Yes

Are sufficient details of the methods provided to allow replication by others?

Yes

Are the datasets clearly presented in a useable and accessible format?

Not applicable

Reviewer Expertise:

Biostatistics, observational epidemiology and clinical trials

I confirm that I have read this submission and believe that I have an appropriate level of expertise to confirm that it is of an acceptable scientific standard.

## References

[1] : Comparison of methods for predicting COVID-19-related death in the general population using the OpenSAFELY platform. *Diagn Progn Res* .2022;6(1) : 10.1186/s41512-022-00120-2 6 10.1186/s41512-022-00120-2 35197114 PMC8865947

# Reviewer report: Reviewer response for version 2

**DOI:** 10.21956/wellcomeopenres.25956.r115736

**Published:** 2024-12-17

**Authors:** Glen Martin

**Affiliations:** 1 Division of Informatics, Imaging and Data Science, Faculty of Biology, Medicine and Health, Manchester Academic Health Science Centre, University of Manchester, Manchester, UK

Thank you for your detailed response to my previous comments. I think these have all been addressed well, and thank you for clarifying points that I previously misunderstood. I will enjoy reading the results of your study when available/published.

Is the study design appropriate for the research question?

Partly

Is the rationale for, and objectives of, the study clearly described?

Yes

Are sufficient details of the methods provided to allow replication by others?

Yes

Are the datasets clearly presented in a useable and accessible format?

Yes

Reviewer Expertise:

Statistics; prediction modelling

I confirm that I have read this submission and believe that I have an appropriate level of expertise to confirm that it is of an acceptable scientific standard.

# Reviewer report: Reviewer response for version 1

**DOI:** 10.21956/wellcomeopenres.17984.r92733

**Published:** 2024-08-19

**Authors:** Glen Martin

**Affiliations:** 1 Division of Informatics, Imaging and Data Science, Faculty of Biology, Medicine and Health, Manchester Academic Health Science Centre, University of Manchester, Manchester, UK

In this protocol, the authors aim to develop and compare predictive performance of risk models for COVID-19 mortality across those CPMs that do, and do not, acknowledge time-varying burden of disease. The hypothesis is that those models that incorporate time-varying disease information will suffer less from calibration drift and will allow risks to be updated with time.

 Overall, the protocol is clear and has very clear aims/objectives. The methods are appropriate to address these aims, and the planned internal-external validation is strong. I particularly liked the planned PPIE activities around people’s perception of risk models, particularly those with time-varying elements. Another strength is the size of the data, which will facilitate some of the quite complex modelling that is planned.

 I do have several comments that I list below in the order of which occurred to me when reading the paper.  I have reviewed this paper from a statistical/risk modelling perspective; I cannot comment on the potential clinical implications or considerations of the planned work.

1. Introduction: the authors rightly highlight that most prediction models assume the underlying data is time invariant, leading to calibration drift (i.e., the model coefficients are time-invariant). Common solutions to this are – as the authors state – to periodically update the model or apply dynamic modelling methods (i.e., to update the beta coefficients through time). However, this is subtly different to the motivation of the current study: to update an individual’s predictions through time by incorporating time-varying covariates (to provide “updated risk predictions as the underlying prevalence of infection changes”). There, the beta coefficients don’t necessarily need to change to allow this (although I realize that in taking a landmarking approach, the coefficients of course will in each landmark sub-model). I thought this subtle – but in my view important – distinction could be highlighted more clearly in the introduction.
2. Model development design A: The following sentences are unclear and appear contradictory: “Note that censoring will not occur at death due to non-COVID causes, because the sub-distribution hazard is the target. The only censoring event in our cohort study is the competing event of death due to other causes, thus the sub-distribution hazard can be estimated by simply not censoring participants at the competing event” I agree with handling of competing risks appropriately, but how is death due to non-COVID causes different to death due to other causes?
3. Model development design A: the authors describe that due to the large number of patients, a sampling approach will be taken. However, the approach taken will directly impact the prevalence of the outcome through time (the handling of which is an aim of the study). Specifically, by sampling all COVID-19 death cases and a random subset of non-COVID-19 death cases (in a ratio of 40:1 as stated by the authors) the prevalence of the outcome is impacted and won’t match the observed prevalence. By definition, the model will then be mis calibrated. Why not just randomly sample from all ~17 million (can still stratify by age if the authors wish, but not by outcome)?
4. Model development design B: same comment as previous re. sampling approach. Moreover, why not just simply use the same individuals randomly selected in design A, so that all models are fitted (and validated) upon the same data?  Given those sampled into design A, then the landmark windows (Fig 1) can then be defined for those individuals. I don’t understand why a different sampling scheme is needed specifically for design B. Please can the authors clarify my misunderstanding.
5. Candidate predictors and variable selection: the authors go to great lengths in the protocol outlining the choice of candidate predictors in terms of a “range of mechanisms driving associations”. However, as the authors identify, there are over 500 COVID-19 models that exist (they cite the BMJ systematic review), many of which are developed for the target population (general population) and for the outcome (COVID-19 death) the authors consider (e.g., the model developed in reference 13 that the authors cite would seemingly match their target population and outcome). As such, its not clear to me why the authors haven’t simply based the underlying model (predictors and functional form) on an existing model, but of course re-fit the model using the different methods that the authors wish to explore and compare. Afterall, I understand that the aim of the study is not necessarily to develop a new model for clinical use, but rather to compare different methods of time-varying modelling in CPMs. This detailed consideration of variable selection suggests that the aim is to develop a new risk model for use. If so, then the aim should be written as such.
6. Following my previous comment, the approach to variable selection is quite confusing to me. The authors describe taking a 4% random sample of the eligible cohort (which I understand is a different sample to their case-cohort sampling described earlier in the protocol) and perform variable selection therein. Its totally unintuitive to me why this is needed. The authors have described in an earlier section the approach to randomly sampling the cohort for design A – why not just model (including variable selection) in that sample? And why 4%? As above, the authors could just avoid all this unnecessary complication to study design by basing their underlying model on prior work. Especially since these complications are not relevant to answering the study research question (does adding in time-varying information about disease burden improve calibration). Similar comment to variable selection in design B (where the proposal is to use 3%).
7. Sample size calculations – why did the authors assume a logistic regression model was being fit, when they are fitting a range of time-to-event models? Sample size criteria exist for time-to-event.
8. Model performance – the Hosmer-Lemeshow test is a weak form of calibration assessment. I agree with the calculation of calibration intercept and slope, but the authors might like to replace H-L test with a flexible calibration plot. See Van Calster B *et al* (2016 ^1^ )

Overall, an interesting study, with strong aim and hypothesis, but some design choices need further explanation or consideration as appear overly complex for the intended aim. I hope the authors find my comments constructive and useful.

Is the study design appropriate for the research question?

Partly

Is the rationale for, and objectives of, the study clearly described?

Yes

Are sufficient details of the methods provided to allow replication by others?

Yes

Are the datasets clearly presented in a useable and accessible format?

Yes

Reviewer Expertise:

Statistical methods for risk prediction modelling

I confirm that I have read this submission and believe that I have an appropriate level of expertise to confirm that it is of an acceptable scientific standard, however I have significant reservations, as outlined above.

## References

[1] : A calibration hierarchy for risk models was defined: from utopia to empirical data. *J Clin Epidemiol* .2016;74: 10.1016/j.jclinepi.2015.12.005 167-76 10.1016/j.jclinepi.2015.12.005 26772608

# Response

**Published:** 2024-12-11

**Authors:** Elizabeth Williamson

**Affiliations:** London School of Hygiene & Tropical Medicine, UK

Thank you for taking the time to review this protocol. The comments were helpful, constructive and quite thought-provoking at times. We apologise for the long delay in responding, which was caused by the primary author being on maternity leave.

 1) Your point about updating coefficients versus modelling time-varying functions of the burden of infection is a good one, and not something that we had really touched on in our manuscript. We have added the following to the introduction: “This aim is subtly different to that of updating models over time. In model updating, the coefficient values in the model are updated to reflect the more recent context. In our approach, (some of) the coefficients are time-varying functions, thus while their values will change over time, the parameter values of those time-varying functions will not.”

 2) Our description of the approach to non-COVID deaths was worded clumsily. We have replaced these two sentences with: “The sub-distribution hazard for COVID-19 death is estimated by not censoring participants if they experience the only competing event (death due to non-COVID causes).”

 3) Regarding our choice of case-cohort sampling approach: Information relating to risk tends to lie largely in participants who experience the outcome. This is often a very small fraction of the population. Random sampling would greatly reduce the information available with which to predict risk. Stratifying the sampling by age would help a little, but would likely lead to very inefficient risk prediction in all but the very oldest groups. Case-cohort sampling is not common in risk-prediction modelling but it is a well established approach in epidemiology more generally. Sampling weights are used to correct for the outcome-dependent sampling. In particular, we applied Barlow weights which weight participants in the subcohort by the inverse of the sampling fraction. Muhlenbruck et al (J Clin Epi, 2017) explore the performance of various weighting schemes in case-cohort studies for estimation of absolute risks. They find that Barlow weights lead to very good agreement of absolute risks and measures of calibration and discrimination in comparison with the full cohort. Under this weighting scheme, therefore, it is incorrect to say that the model is by definition mis-calibrated.  We have added the following text: “This sampling strategy was selected because information relating to risk tends to lie largely in participants who experience the outcome, which is a very small fraction of the overall population here. Random sampling would greatly reduce the information available with which to predict risk. Case-cohort sampling, with the weights we propose to use (Barlow), has been shown to lead to very good agreement of absolute risks and measures of calibration and discrimination in comparison with use of the full underlying cohort. (Muhlenbruck et al, JClinEpi, 2017)”

 4) Our rationale for the sampling strategy for variable selection was perhaps not hugely clear. Design B required a smaller sampling fraction due to computational considerations, since the stacking of different landmark studies increases the size of the data being used for the lasso. We also did not want to re-use data from different landmark studies for the same people, to minimise one participant’s influence on variable selection, so we selected different sets of participants for the different landmark studies used in variable selection.  We could have tried to find a sampling strategy that maximised overlap of the participants whose data was used for variable selection in Design A and B, but this seemed - at the time - to be more complex than just using random samples for each.

 5) You query our rationale for incorporating variable selection within our comparative study, on the grounds it would be a cleaner comparison if we restricted it to a fixed set of model variables from an existing model. It is very possible that many variables which are strong predictors of risk of COVID-19 death have much weaker relationships with risk once the changing pattern of infection over time is taken into account. Variables such as deprivation, ethnicity and region, for example, might have quite different associations with risk in models that do and do not take the burden of infection into account. Therefore, the variable selection is intrinsically bound up in the approach taken to the changing pattern of risk over time. This is the rationale for including a variable selection procedure within our comparison study. In addition, although many risk prediction models had been developed at the time, the review of these models mentioned by the reviewer concluded that “almost all pubished prediction models are poorly reported, and at high risk of bias” (Wynants, BMJ, 2020). Further, almost all of these models were developed in very different settings to ours, putting the generalizability of the model to our setting into question. The one model, QCOVID (Clift, BMJ, 2020), that was developed in similar data was not available for re-use at the time the current protocol was written. Finally, not all variables used in QCOVID were available in the OpenSAFELY database. Overall, there was no suitable model in existence that we could have directly applied and we felt it was important to consider the inter-relationship between variable selection and the modelling approach taken to changing infection burden by including a variable selection process within each modelling approach. We have added a brief explanation of this to the manuscript: “While the focus of this study is on the impact of explicitly modelling the changing burden of infection over time, some of this impact may occur through changes in the variables that are selected to be in the model. Variables that strongly predict risk in models that do not account for the changing burden of infection over time may have a weaker, or non-existent, relationship with risk once this aspect is explicitly modelled. Therefore, variable selection is intrinsically bound up in our comparison of interest. Below, we describe our variable selection strategy.:”

 6) Our initial plan was to perform variable selection within the case-cohort study. However, the re-weighting required to correct for case-control sampling was not compatible with our statistical software implementations of lasso model selection. Therefore, we were unable to perform variable selection within the case-cohort, nor was there sufficient computational resource to perform variable selection within the entire cohort. The number of 4% was the largest sample possible while limiting use of computational resources to an acceptable level. We have added a comment about this to the manuscript: “Variable selection will be performed, for approach A, within a 4% random sample of the whole eligible cohort. The sampling fraction was chosen as the largest fraction possible while keeping computational resources within the limits that will be available for this project.”

 7) For design B, the 28-day window means that logistic and time-to-event models are almost indistinguishable and thus power calculations for logistic are reasonable. For design A, it is likely that there is some degree of approximation in the sample size calculations. However, we decided to keep the same sample size calculations for both designs for simplicity.

 8)  Thank you for noting that our choice of Hosmer-Lemeshow test is not optimal for calibration assessment and suggesting flexible calibration plots instead. The study results do include flexible calibration plots (Williamson et al, Diagn Progn Res, 2022). We agree with your suggestion, but given the protocol and study are now publicly available we would prefer to leave our planned approach as it initially was for transparency.    

**References:**

 Mühlenbruch, Kristin et al. Multiple imputation was a valid approach to estimate absolute risk from a prediction model based on case–cohort data. *Journal of Clinical Epidemiology*, Volume 84, 130 – 141, 2017. https://www.jclinepi.com/article/S0895-4356(17)30057-4/fulltext

 Wynants L, Van Calster B, Collins G S, Riley R D, Heinze G, Schuit E et al. Prediction models for diagnosis and prognosis of covid-19: systematic review and critical appraisal BMJ 2020; 369 :m1328 doi:10.1136/bmj.m1328 

 Clift AK, Coupland CA, Keogh R, *et al.*: QCOVID a living risk prediction algorithm for risk of hospitalisation and mortality from COVID-19 in adults: national derivation and validation cohort study. *BMJ.*

 The OpenSAFELY Collaborative., Williamson, E.J., Tazare, J. *et al.* Comparison of methods for predicting COVID-19-related death in the general population using the OpenSAFELY platform. *Diagn Progn Res* **6**, 6 (2022). https://doi.org/10.1186/s41512-022-00120-2

# Reviewer report: Reviewer response for version 1

**DOI:** 10.21956/wellcomeopenres.17984.r40946

**Published:** 2020-10-30

**Authors:** Lucy Teece

**Affiliations:** 1 Department of Health Sciences, University of Leicester, Leicester, UK

The article reports the protocol for a planned study to develop, validate, and compare prediction models for COVID-19 death at 28 days. This important study will investigate statistical methods to incorporate changes in infection burden over time, and evaluate whether these methods can improve the predictive ability of COVID-19 risk prediction models. To my knowledge, no studies have been published that investigate the impact of incorporating time-varying predictive factors into COVID-19 risk prediction models, and the article content is novel. The authors clearly articulate the rationale for the planned study, including the importance of accurate prediction models to facilitate appropriate risk management of individuals, and the impact the underlying infection burden is likely to have on changes in risk. I commend the author’s use of prediction model specific reporting guidelines (TRIPOD), sample size calculation, and risk of bias tool (PROBAST) to ensure appropriate components of the planned study have been included in the article, appropriate sample size calculation, and assessment of bias has been conducted. The article is well written, the planned study poses an interesting and original question, and I look forward to reading the study findings once completed.

 I present a short list of points to address:

1. Authors primarily use the term “sex”, but refer to missing “gender” when discussing eligibility criteria; it might help the reader to keep this consistent.
2. Within the *Objectives,* the authors state the use of Cox proportional hazards model. However, in the *Model development design*, they discuss competing risks and the aim to attain subdistribution hazards. So the model fitted will be a Fine and Gray proportional subdistribution hazard model: https://www.tandfonline.com/doi/abs/10.1080/01621459.1999.10474144. ^1^

Is the study design appropriate for the research question?

Yes

Is the rationale for, and objectives of, the study clearly described?

Yes

Are sufficient details of the methods provided to allow replication by others?

Yes

Are the datasets clearly presented in a useable and accessible format?

Not applicable

Reviewer Expertise:

Medical statistics with a focus on prognostic modelling, competing risks methodology, and use of electronic health record data

I confirm that I have read this submission and believe that I have an appropriate level of expertise to confirm that it is of an acceptable scientific standard.

## References

[1] : A Proportional Hazards Model for the Subdistribution of a Competing Risk. *Journal of the American Statistical Association* .1999;94(446) : 10.1080/01621459.1999.10474144 496-509 10.1080/01621459.1999.10474144

# Response

**Published:** 2024-12-11

**Authors:** Elizabeth Williamson

**Affiliations:** London School of Hygiene & Tropical Medicine, UK

Thank you for taking the time to review this protocol. We apologise for the long delay in responding, which was caused by the primary author being on maternity leave. Two specific responses to your comments:

 1) Thank you for noting the discrepancy in our use of the words “sex” and “gender”. We’ve changed to “sex” throughout.

 2) Also thank you for pointing out our inconsistent description of the proportional hazards model. We have clarified within the Objectives that a Fine and Gray proportional sub-distribution hazard model was used.
